# Mobile caecum as a content in recurrent incisional hernia an incidental finding: A rare case report

**DOI:** 10.1016/j.ijscr.2021.105672

**Published:** 2021-02-22

**Authors:** Chinniahnapalaya Pandurangaiah Hariprasad, Rohit Gupta, Anil Kumar, Deepak Kumar Jha, Shiv Kishor, Shiv Shankar Paswan

**Affiliations:** a Department of General Surgery, All India Institute of Medical Sciences, Patna, India; b Department of Trauma & Emergency (Gen Surgery), All India Institute of Medical Sciences, Patna, India

**Keywords:** Mobile caecum, Intraperitoneal on lay mesh repair, Caecopexy

## Abstract

**Introduction:**

Recurrence in ventral hernia after laparoscopic repair is less as compared to conventional approach. Mobile caecum as a content of ventral hernia is a very rare entity. Standard treatment for mobile cecum is caecopexy using lateral peritoneal flap.

**Case report:**

A 40-year-old obese female, homemaker by occupation with a history of incisional hernia 2 year back and treated with intraperitoneal on lay mesh repair presented with swelling in the left lower abdomen for past 6 months. Radiological investigations revealed defect in left lower anterior abdominal wall with protruding bowel loops. Urgent exploratory Laparotomy revealed mobile segment of ileocecal junction in the hernial sac cavity. Caecopexy for the mobile caecum was done.

**Discussion:**

Mobile caecum is due to embryological failure of fusion of right colonic mesentery with lateral peritoneal wall. Pre-operative diagnosis of mobile caecum is difficult to establish unless it presents as caecal volvulus Caecopexy using the lateral peritoneal flap is the standard of care.

**Conclusion:**

Mobile caecum can surprise the attending surgeon as a content of ventral hernia. Caecopexy using lateral peritoneal flap is the treatment of choice in all with a mobile caecum.

## Highlights

• Mobile caecum as a content of hernial sac in recurrent incisional hernia is a very rare entity.
• As per the available literature this is the first case of mobile caecum as content of recurrent incisional hernial.
• Caecopexy using the lateral peritoneal flap is the treatment of choice for mobile caecum.

## 1 Introduction

Laparoscopic approach for the repair of ventral hernia is accepted as the standard of care. Recurrence after laparoscopic repair is less as compared to open conventional approach [1]. Failure of fusion of caecum, terminal ileum and right colon along with its mesentery to the posterior parietal peritoneal wall is defined as mobile caecum [2]. Mobile cecum is an uncommon cause of right lower abdominal pain and incorrectly perceived as appendicitis. We present a rare case of 40-year-old obese female with recurrent failed laparoscopic intraperitoneal on lay mesh repair (IPOM) and mobile caecum as a content. This case report is in line with SCARE criteria [3].

## 2 Case report

**Fig. 1:**
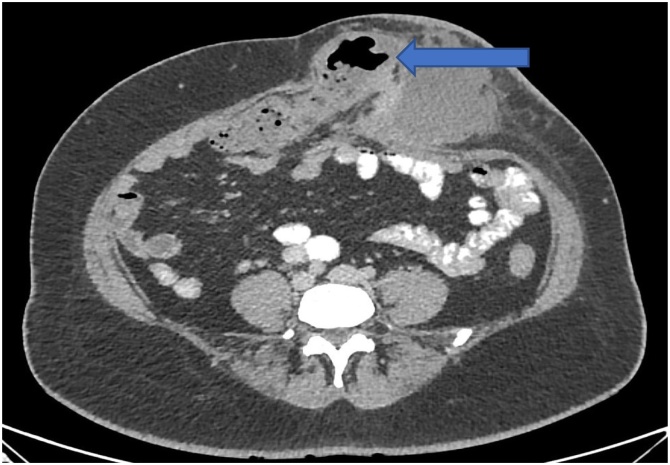
CECT Abdomen with arrowhead pointing at the defect in anterior abdominal wall with bowel loops protruding through it.

**Fig. 2:**
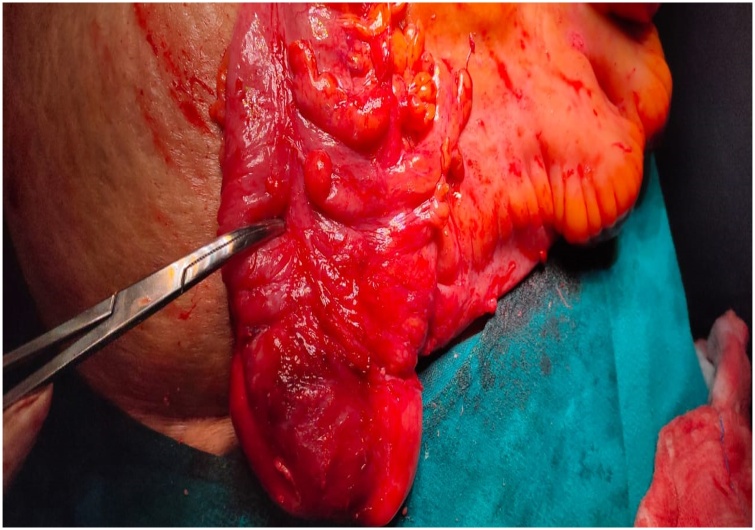
Forceps pointing towards the mobile caecum removed from the hernial sac.

A 40-year-old obese female homemaker by occupation presented to ED (Emergency department) of our tertiary care center with history of swelling over left lower abdomen for past 6 months. Swelling was associated with dull aching pain which has aggravated in past 10 days. Patient also had recurrent episode of vomiting and fever not relived by antipyretics. She had a history of previous lower segment caesarean section 14 years ago following which she developed incisional hernia after 2 years. Patient underwent intraperitoneal on lay mesh repair (IPOM) a year ago. She was a known case of type 2 diabetes mellitus and hypothyroidism on regular oral medications. No significant family and psychosocial history. She was hemodynamically stable with a pulse rate of 90 beats per minute, blood pressure 110/70 mm of Hg, Spo2 99% at room air, respiratory rate 22 cycles per minute and temperature of 100 Fahrenheit. Physical examination revealed a soft tender swelling of 5 × 7 cm in the left hypogastric region, skin over the swelling was erythematous with local raise of temperature. Ultrasonography of the abdomen revealed a defect of about 3 cm in the anterior abdominal wall on left side of surgical incision through which bowel loop was seen herniating into the subcutaneous plane, few pockets of loculated collections along with septation noted. Contrast enhanced computed tomography revealed a defect of size 4 × 3 cm in the anterior abdomen wall in hypogastric region left to the midline with bowel loops protruding through it [Fig. 1]. All investigations were within the normal limit except total leucocyte count which was 15.40 × 10^3^ per μl. Urgent exploratory laparotomy was planned by a team consisting of an additional professor of general surgery with a 12-year experience. Intraoperative findings revealed a freely mobile segment of bowel loop with ileocecal junction [Fig. 2] and small bowel trapped in hernial cavity. Meticulous adhesiolysis and retrieval of the gut segment done along with the fixation of caecum in lateral abdominal wall by caecopexy, large collection of pus found and drained through separate incision, thorough peritoneal toileting done. Post-operative period was uneventful and the patient was discharged on day 12. Regular follow up of the patient was done at 1 month, 3 month and 6 months and she was doing well at all visit.

## 3 Discussion

Abdomen is a pandora’s box, hidden uncertainties and incidental findings bewilder every surgeon and add to the literature, one such finding is mobile caecum presenting as a content of ventral hernia. So far only two cases have been reported with caecal volvulus as a content in ventral hernia [4,5]. Mobile caecum can be attributed to embryological failure in fusion of right colonic mesentery with lateral peritoneal wall. Common symptoms related to mobile caecum syndrome includes colicky pain in right lower abdomen along with abdominal distension and inability to pass flatus and stools. Pre-operative diagnosis of mobile caecum is difficult to establish unless it presents as caecal volvulus. As in our case CECT revealed bowel loops protruding through the defect which to our utter surprise was a mobile caecum revealed intraoperatively. Recurrence after laparoscopic IPOM repair may be attributed to various causes. High Body mass index, rectus abdominis diastasis, improper closure of anterior rectus fascia and mesh material related factors are among the few known causes [6]. Proper inspection of entire abdominal cavity and its contents is of paramount importance during any laparospic surgery, which may sometimes surprise the surgeon with unexpected findings like mobile caecum which can be treated then and there avoiding morbidity to the patient like index case. Surgical technique of choice for fixation of mobile caecum is caecopexy using lateral peritoneal flap as described by Dixon and Meyers [7]. However, laparoscopic caecopexy is attaining popularity and may be considered as a standard treatment option. A prospective study conducted by Garude k et al. [8] in patients with right lower abdominal pain planned for appendectomy revealed 8 patients with mobile caecum among 110 study subjects all of which underwent caecopexy with satisfactory outcome. As per Garude k et al. the satisfactory outcome was assessed by complete abdominal wound healing at the time of discharge, sutures removal was done during 8th to 10th post-operative day and thereafter patient was followed up for gastrointestinal complaints twice monthly for first two months and thereafter thrice monthly. In our case the outcome was assessed by complete wound healing, no surgical site infection and regular bowel habit. On 12th Post-operative day, the sutures were removed and patient was doing well. Regular follow up of the patient was done at 1 month, 3 month and 6 months with all normal noted parameters including normal bowel habit, no pain abdomen with normal ultrasonography report.

## 4 Conclusion

Mobile caecum can surprise the attending surgeon as a content in ventral hernia. Bowel viability should be ascertained before planning a definitive procedure. Caecopexy using lateral peritoneal flap is the treatment of choice in all with a mobile caecum.

## Declaration of Competing Interest

The authors report no declarations of interest.

## Funding

All authors have no source of funding to disclose.

## Ethical approval

There is no ethical approval was obtained as it’s a case report.

## Consent

Written informed consent was obtained from the patient for publication of this case report and accompanying images. A copy of the written consent is available for review by the Editor-in-Chief of this journal on request.

## Authors contribution

Dr Hariprasad C P: Writing the paper.

Dr Rohit Gupta: Study concept.

Dr Anil Kumar: Operated, revised and edited the manuscript.

Dr Deepak Kumar Jha: Operated the Case.

Dr Shiv Kishor: Review of the literature.

Dr Shiv Shankar Paswan: Critically analysed.

## Registration of research studies

Not Applicable.

## Guarantor

Dr Anil Kumar.

## Provenance and peer review

Not commissioned, externally peer-reviewed.

## References

[1] Heniford B.T., Park A., Ramshaw B.J., Voeller G. (2003). Laparoscopic repair of ventral hernias: nine years’ experience with 850 consecutive hernias. Ann. Surg..

[2] Makama J.G., Ahmed A., Ukwenya Y., Mohammed I. (2009). Mobile caecum and ascending colon syndrome in a Nigerian adult. Ann. Afr. Med..

[3] Agha R.A., Franchi T., Sohrabi C., Mathew G., for the SCARE Group (2020). The SCARE 2020 guideline: updating consensus surgical CAse REport (SCARE) guidelines. Int. J. Surg..

[4] Reznichenko A.A., Macaluso F., Zulim R. (2015). Cecal volvulus in giant ventral hernia. Int. J. Surg. Case Rep..

[5] Ting Y.Y., Farfus A., Trochsler M. (2020). Caecal volvulus in an incisional hernia. J. Surg. Case Rep..

[6] Langer C., Neufang T., Kley C., Liersch T., Becker H. (2001). Central mesh recurrence after incisional hernia repair with Marlex--are the meshes strong enough?. Hernia.

[7] DIXON C.F., MEYER A.C. (1948). Volvulus of the cecum. Surg. Clin. North Am..

[8] Garude K., Rao S. (2013). Mobile cecum: an incidental finding. Indian J. Surg..

